# Enzymatic Transesterification of Atlantic Salmon (*Salmo salar*) Oil with Isoamyl Alcohol

**DOI:** 10.3390/ma16031185

**Published:** 2023-01-30

**Authors:** Milda Gumbytė, Violeta Makareviciene, Egle Sendzikiene

**Affiliations:** Agriculture Academy, Vytautas Magnus University, K. Donelaicio Str. 58, LT-44248 Kaunas, Lithuania

**Keywords:** salmon oil, transesterification, isoamyl alcohol, optimization, response surface methodology

## Abstract

In this experimental study, biodiesel was synthesized from the salmon oil using the Lipozyme^®^RM IM (Bagsværd, Denmark) as a biocatalyst. Isoamyl alcohol was used as an acyl acceptor in the transesterification process. The aim of this study is to select the best process conditions, aiming to obtain the highest transesterification degree that meets the requirements of the EN 14214 standard. Response surface methodology (RSM) was used for statistical analysis and optimization of process parameters. A four-factor experimental design was modelled by central compositional design (CCD) to investigate the effects of biocatalyst concentration, isoamyl alcohol-to-oil molar ratio, temperature, and duration on transesterification degree. It was determined that the optimal parameters for biodiesel synthesis were the following: an enzyme concentration of 11% (wt. of oil mass); a process temperature of 45 °C; a process duration of 4 h; and an alcohol-to-oil molar ratio of 6:1. The transesterification degree of biodiesel reached 87.23%. The stepwise addition of isoamyl alcohol during the transesterification process further increased the degree of transesterification to 96.5%.

## 1. Introduction

Rapid economic development and the growing global population increases energy demand worldwide [1,2]. Scientists predict that, by 2030, global oil consumption will reach 118 million barrels per day, and oil reserves will be exhausted by 2060 [3,4,5]. The economic development of a country depends on how much the energy needs are met, so the demand for fuel is growing [6]. Despite increasing environmental pollution due to greenhouse gas emissions, diesel engines are widely used in industrial, agricultural, and transportation sectors [5,7,8]. Due to dwindling fossil fuel resources, fluctuating oil prices, increasing environmental threats, and stricter emission standards, the use of renewable energy sources for fuel production remains an important future prospect [9]. An alternative to mineral diesel is biodiesel, which reduces the emissions of harmful components [5,7,8]. Among all renewable fuels, biodiesel has received a lot of attention because it is an environmentally friendly fuel, less toxic than diesel fuel, and it biodegrades faster, reducing the negative environmental affects [1,3,10]. Properly produced biodiesel, i.e., after small modifications of the triglyceride molecule using alcohol and various catalysts, can be used either pure or mixed with mineral diesel in most current diesel vehicles [7,11]. The quality requirements for this automotive diesel substitute can be found in the standards. The current European standard for 100% fatty acid methyl esters (RRME) is EN 14214. In the instance that the produced biodiesel does not meet the requirements of the standard, it can be used in boilers to produce heat (depending on its properties), or various raw materials can be mixed with each other to improve the quality of the feedstock and, at the same time, the quality of the produced biodiesel [11].

Typically, biodiesel is produced through a series of alcoholysis reactions, in which triglycerides present in oil or fat are converted to diglycerides, monoglycerides, and finally to monoalkyl esters of fatty acids (i.e., biodiesel) and glycerol. The alcoholysis reaction is a reversible reaction, so Le Chatelier’s principle can be used to control the results of this reaction, e.g., to increase the yield of products. In addition to lowering the temperature, increasing the concentration of reactants and removal of reaction products can shift the reaction to the right [12]. In addition to the conventional oil or fat used for biodiesel production, alternative sources based on waste or low-quality oil and fat are of increasing interest, as they are cheap raw materials [10,13].

The use of low-quality fish oil that does not meet the quality requirements applied by the pharmaceutical and food industries has become a promising alternative for the production of biodiesel, especially considering the fact that about 90 million tons of fish waste are generated annually due to the increasing consumption of fish worldwide, which promotes the development of the fishing industry [13,14].

The processing of fish waste into biodiesel not only helps to supply sustainable fuel, but also meets the goals of the term “circular bioeconomy”, which appeared in 2015 and has been increasingly used in scientific literature since 2016. The circular bioeconomy, based on the “zero waste” approach, promotes the impact of the process on the environment by reusing, recycling, and utilizing the biogenic waste generated during the process. In the food industry, for example, in the fish industry, a lot of biogenic waste is generated, which is considered as a potential raw material for the development of a circular bioeconomy. As indicated, hybrid renewable energy systems (HRES) are recommended for power generation in coastal areas and remote islands, due to their distance from the national power grid. As the fish industry is one of the main industries in these regions, the production of biodiesel from fish processing waste and its use as fuel can promote the sustainability of electricity generation. In addition, the production of biodiesel from fish waste is more environmentally friendly than the production of biodiesel from vegetable oil. Specifically, the total environmental impact of 1 L of biodiesel from vegetable oil (soybean, rapeseed and palm blend, EcoInvent data) is 21.1 EUR, compared to 4.30 EUR associated with 1 L of biodiesel from fish oil, i.e., the overall environmental impact is reduced by approximately 400% [15,16].

The production, processing, and preservation of fish generates large amounts of waste, with a proportion of the total fish catch being released as processing waste, such as heads, fins, skin, carcasses, offal, and viscera. A more comprehensive utilization is achieved by processing the remains into fishmeal and fish oil. Studies have shown that the amount of fish oil extracted from fishmeal residues varies greatly, from 1.4% to 40.1%, depending on the fish species, organ, and season [4,17,18,19]. Fish oil obtained from various waste sources is proposed for use in biodiesel production by transesterification with methanol, using an alkaline catalyst [13,20]. Given that fish oil contains more than 2% of free fatty acids, transesterification using alkaline catalysts produces soap, which hinders the separation of biodiesel from glycerol and reduces the yield of the final product. Therefore, direct transesterification of this oil using alkaline catalysis is difficult. In that case, free acid esterification using acid catalysts is usually performed prior to conventional transesterification. However, with acid catalysts (sulphuric, sulfonic, or hydrochloric acids), the reaction rate is much lower, and they are very sensitive to water content. From this point of view, enzymatic catalysis becomes a good alternative, because enzymes are more resistant to oil quality fluctuations, require less energy, and can catalyze esterification and transesterification reactions simultaneously, eliminating the need for an additional step of esterification of free fatty acids [20,21]. However, it is a more ecological and environmentally friendly method: during the reaction, further processing costs are reduced; the amount of water in the raw material does not affect the quality of the final product; and the process takes place under mild conditions [13,18,20,21]. Lipase is usually used as a catalyst during the enzymatic transesterification process. In the past decade, enzyme immobilization technology has been widely explored, due to its great potential for large-scale continuous operation, reusability, and stability. In addition, properly performed immobilization of lipases can improve many enzyme properties, such as enzyme activity, stability, selectivity, or specificity. It can also expand the range of conditions under which the enzyme can be used, increasing resistance to chemicals or inhibitors [22,23].

The most commonly used acyl acceptors in biodiesel synthesis are alcohols. Most studies on biodiesel production have been conducted using methanol [18,24,25], ethanol [19,26], and butanol [7,21]. Some researchers are conducting transesterification studies with longer and branched chain alcohols [27]. It was established that the transesterification process becomes more complicated as the alcohol chain increases. When enzyme preparations are used as catalysts, the greatest negative effect was observed for transesterification using methanol, which poisons the enzymes and makes them lose their catalytic activity. Information on the use of isoamyl alcohol in the synthesis of biodiesel from fish oil could not be found in the scientific literature. This alcohol was chosen for our research as an alternative to conventional methanol due to the possibility of synthesizing it using more environmentally friendly methods, avoiding chemical synthesis from natural gas, which is used in methanol synthesis. The research results show that isoamyl alcohol can be obtained not only by chemical synthesis, but also by applying environmentally friendly biotechnological methods. It can be obtained by enzymatic methods from 3-methylbutyryl-CoA (isovaleryl-CoA). Isoamyl alcohol could be synthesized by the yeast Saccharomyces cerevisiae, using leucine as a raw material [28]. This alcohol has a low eco-tox profile and is therefore widely used in various industries.

The aim of this study was to optimize the transesterification process of salmon oil with isoamyl alcohol using response surface methodology (RSM). Multiple regression and correlation analysis was applied to evaluate the effects of four independent factors on biodiesel yield. The effect of interaction of four independent variables—temperature, molar ratio of isoamyl alcohol to oil, enzyme concentration, and process duration—on the degree of transesterification of biodiesel was evaluated, and the optimal conditions, under which the maximum degree of transesterification is obtained, were determined.

## 2. Materials and Methods

### 2.1. Materials

Atlantic salmon (*Salmo salar*) oil was obtained from JSC “Akvatera LT” (Lithuania). It was kept in a refrigerator at 4 °C during the experimental period. *Candida antarctica* B lipase immobilized on Lewatit VP OC 1600 (Lipozyme^®^435; 10,000 U/g, Bagsværd, Denmark), *Rhizomucor miehei* lipase immobilized in beads from macroporous anion exchange resins (Lipozyme^®^RM IM; 20,000 U/g Bagsværd, Denmark) and *Thermomyces lanuginosus* lipase immobilized on silica gel (Lipozyme^®^TL IM; 50,000 U/g) were kindly donated by a Novozymes (Bagsværd, Denmark) representative in Lithuania JSC “Biopolis”. All solvents and reagents used for synthesis and analysis according to the requirements of standards were of chromatographic or analytical grade and obtained from Sigma-Aldrich.

### 2.2. Methodologies for the Study of Oil and Biodiesel Properties

The following properties of the oil were determined: acidity and acid number (according to EN ISO 660); moisture content (according to EN ISO 665); iodine number (according to ISO 3961); kinematic viscosity at 40 °C and density at 15 °C were determined using a SVM™ 3000 Stabinger Viscometer™ from Anton Paar (Graz, Austria). Color parameters are determined (L*, a*, b* values by CIE L*a*b* method) according to Pando et al.’s methodology [29]. The analysis of fatty acid composition was based on the requirements of ISO 5508 and ISO 5509 standards. The molar mass of salmon oil was determined according to Cardoso et al.’s methodology [26].

The quality of biodiesel was assessed according to the requirements of the European biodiesel standard EN 14214. The following parameters were determined: acid number (according to EN 14104); kinematic viscosity at 40 °C (according to EN ISO 3104) using SVM™ 3000 Stabinger Viscometer™ from Anton Paar; density at 15 °C (according to EN ISO 3675) using SVM™ 3000 Stabinger Viscometer™ from Anton Paar; ester content by GC method according to standard EN 14103; iodine number (according to EN 14111); mono-, di- and triglycerides content (according to EN 14105); free and total glycerol content (according to EN 14105; EN 14106); calorific value (according to DIN 51900). Gas chromatographic analysis was performed using a gas chromatograph, Perkin Elmer Clarus 500 (GC/FID), with a Split/Splitless injector. The chromatograph was connected to a capillary column (ZEBRON ZB-FAME (30 × 0.2 mm × 0.25 μm)). Gas carrier (hydrogen) pressure—constant, 90 kPa, split—1:100. Chromatograph temperature program: initial analysis temperature 100 °C, the temperature of the included sample is maintained for 2 min, then increased to 240 °C at a rate of 3 °C/min. After reaching 240 °C, the temperature is maintained for 5 min. The temperature of the detector is constant—285 °C. As a final result, the arithmetic mean of the results of three tests was taken. The absolute difference did not exceed 1%.

### 2.3. Transesterification of Salmon Oil

Three immobilized industrial lipases (Lipozyme^®^ RM IM, Lipozyme^®^ TL IM, Lipozyme^®^ 435) (Bagsværd, Denmark) were used as biocatalysts in biodiesel synthesis. In the first stage, to select the most efficient lipase, tests were carried out using different lipases under the same process conditions. The transesterification process was performed in a conical flask connected to a condenser, a thermometer with a temperature controller, and a stirrer (at a constant speed of 225 1/min). The conical flask was placed in a glycerol bath to equilibrate the temperature of the mixture. The required content of salmon oil was added to the flask, constantly stirred, and thermostated. After the oil was heated to the required temperature, alcohol and biocatalyst were added to the oil (depending on the amount of oil). After the specified reaction time, lipase was filtered, excess of alcohol was removed with a vacuum rotary evaporator (IKA RV 06-ML 1-B Distilling Rotary Evaporator, Staufen, Germany), and glycerol phase was removed by decantation. The remaining mixture was washed twice with water (10% of the ester content), in order to remove glycerol formed and purify the produced esters, and was dried and analyzed by gas chromatography according to the requirements of the standard EN 14105.

### 2.4. Optimization and Statistical Analysis of the Transesterification Process

The optimization of the transesterification process was carried out in a four-factorial experiment to investigate the effect of isoamyl-alcohol-to-salmon-oil molar ratio (A), reaction temperature (B), biocatalyst concentration (C), and reaction time (D) (Table 1) on the transesterification degree using Central composite design (CCD). The CCD consisted of 27 experimental trials (Table 2) (2 k + 2 k + m, where k is the number of independent variables and m is the number of repeated central points), 16 factorial points (2 k), eight axial points (2 k), and three repeated central points (m = 3). The axial points are distant from the central point by a distance α, the value of which is chosen as practical (1.2). The model uses the transesterification degree (%) response. The program Design-Expert 7.01 (Stat-Ease, Minneapolis) was used to perform variance (ANOVA) and graphic analysis of the obtained data. The obtained experimental data (Table 2) were analyzed using the statistical analysis system (SAS) response surface regression (RSREG) method, which fits the second-degree polynomial model (Equation (1)). The RSREG method used canonical analysis to estimate stationary values for each variable. Using the fitted model, response surface contour plots were constructed for each pair of variables, with the third variable held constant at the estimated stationary point. To validate the model, optimization of the reaction conditions was performed using combinations of independent variables that were not included in the original experimental design.

The experimental data were analyzed using response surface regression procedure that fits a full second-order polynomial model:

(1) $$Y=\beta_{0}+\overset{3}{\underset{i=1}{\sum }}\beta_{i}X_{i}+\overset{3}{\underset{i=1}{\sum }}\beta_{ii}X_{i}^{2}+\overset{2}{\underset{i=1}{\sum }}\;\overset{3}{\underset{j=i+1}{\sum }}\beta_{ij}X_{i}X_{j}$$

where:

*Y*—predicted response,
*X_i_*, *X_j_*—independent variables,
*β*_0_, *β_i_*, *β_ii_* and *β_j_*, *β_ij_*—interaction constant coefficients.

## 3. Results and Discussions

### 3.1. Oil Properties

The properties of salmon oil are presented in Table 3. The studied salmon oil is characterized by a high number of free fatty acids, although acid numbers of oil is within the limits of the oils studied by other Portuguese researchers [4] (from 0.1 to 28.4 mg KOH/g). A high acid number is known to affect oil transesterification using alkaline catalysts, resulting in a significant reduction in product yield, as soap is produced in addition to ester synthesis. Since this study deals with a transesterification reaction using lipase as a biocatalyst, high acidity was not an issue. According to Moreira et al. [30], in the production of biodiesel using enzymatic catalysts, soaps are not formed, which promotes an increase in the reaction yield. The iodine value of fish oil can vary from 88 g l2/100 g to more than 150 g L_2_/100 g [11]. The differences are mainly related not only to the type of fish, but also to the conditions under which the oil was produced.

The tested oil was characterized by green (a* < 0) and yellow (b* > 0) color intensities. The oil studied by Spanish scientists [29] was characterized by red (a* > 0) and yellow (b* > 0) color intensities. The amount of saturated fatty acids found in the studied salmon oil was 20.26%, and the amount of unsaturated fatty acids was 79.76%. In addition, the content of monounsaturated fatty acids in the salmon oil was almost twice that of polyunsaturated fatty acids. The presence of unsaturated fatty acids increases the rate of oxidation. Salmon oil was found to contain 37% more monounsaturated, 27% less saturated and 53% polyunsaturated fatty acids than in the salmon oils studied by a foreign scientist [4].

### 3.2. Selection of Biocatalyst for Transesterification Process

**Figure 1:**
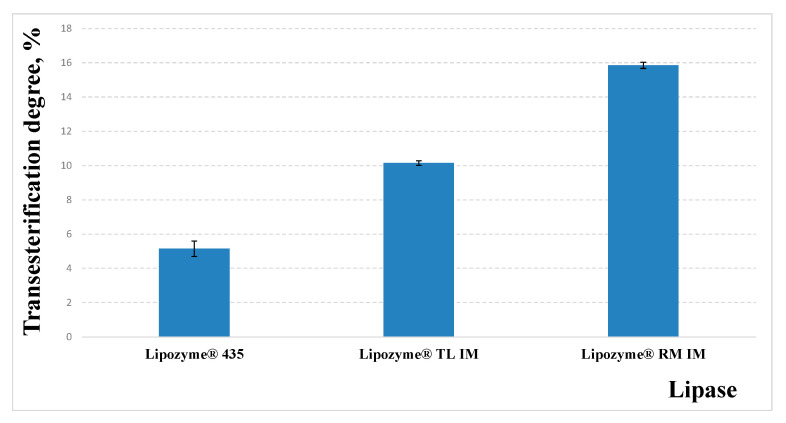
Dependence of transesterification degree on the species of lipases under the same process conditions.

Enzyme catalyst selection plays an important role in the biodiesel synthesis process. Enzymatic catalysts (lipases) are more superior to chemical catalysts, due to their ability to catalyze various triglyceride substrates for biodiesel production, i.e., the specificity of lipases differs for each reaction taking place [13], so it was important to perform evaluation of lipases selectivity for the process of transesterification of salmon oil with isoamyl alcohol. The transesterification process was investigated under the same conditions: the molar ratio of alcohol and oil was stoichiometric (3:1); the reaction temperature was 40 °C, the duration was 4 h; and the enzyme content was 5% of the oil mass. The obtained results are presented in Figure 1.

As can be seen from the obtained results, the catalytic activity of lipases in transesterification reaction is different. At the same amount of enzyme preparation, the reaction catalyzed by Lipozyme^®^ RM IM is almost three times faster than the reaction catalyzed by Lipozyme^®^ 435. Although C. antarctica lipase (Lipozyme^®^ 435) is widely used for biodiesel synthesis studies [31], unfortunately, in our case, this enzyme preparation was not very effective after 4 h. The transesterification degree was only 5.2 ± 0.45%; however, using R. miehei lipase (Lipozyme^®^ RM IM), a transesterification degree of 15.87 ± 0.18% was observed. Considering the fact that the enzyme preparation Lipozyme^®^ RM IM was the most effective for the alcoholysis reaction, so this lipase was selected for further studies. The same tendency was observed by Park et al. among the seven lipases used in [32] their studies, the immobilized *Rhizomucor miehei* lipase was the most effective.

### 3.3. Response Surface Analysis

It is known that the most important parameters affecting the efficiency of biodiesel production are the molar ratio of alcohol to oil (A), temperature (B), the amount of biocatalyst (C), and reaction time (D). In order to investigate the interaction of independent variables (the overall effect of these factors), the experiments were performed by changing the physical parameters using the experimental design (Table 2). By applying multiple regression analysis to the obtained data, the experimental results of the factorial central composite design were fitted to a quadratic polynomial equation (1). The resulting adjusted model for the synthesis of isoamyl alcohol esters is given in Equation (2).

Y = −197.78 + 5.77 A + 9.65 B + 5.07 C + 1.74 D + 0.01 AB − 0.051 AC − 0.066 AD + 0.051 BC + 5.3310^−3^ BD + 0.038 CD − 0.38 A^2^ − 0.13 B^2^ − 0.35 C^2^ +8.8810^−3^ D^2^ (2)

where:

Y—transesterification degree, %;
the alcohol-to-oil molar ratio, mol/mol;
temperature, the catalyst (snail shells) amount, °C;
lipase amount, wt. %;
the process duration, h.

Statistical analysis of the model was performed to evaluate the variance (ANOVA) and test the fit of the empirical model. The ANOVA results fitted to the second-order response surface model by the mean-square method are summarized in Table 4. The coefficients of the response surface model were also estimated as predicted in Equation (2). The significance of each of the coefficients is tested by *p*-values (probability of error value), which also indicate the strength of interaction between each parameter. According to the data presented in Table 4, the *p* value of the model is less than 0.0001, which indicates a high significance in predicting the response values and the fitness of the derived model, and there is only a 0.01% chance that such high F values of the models are due to noises (errors, natural scatter). A high F value (F model = 321.38) with a very low probability value (*p* < 0.0001) indicates the high significance of the constructed model. Evaluation of the discrepancy of the residual errors compared to the theoretical error (F value 1.97) showed that the discrepancy is insignificant. Significances of all coefficients were determined by *p* values and presented in Table 4.

A higher F value and a lower *p* value indicate that the respective parameters are significant. *p*-values “*p* > F” less than 0.05 mean that the model components are significant. For this model, A, B, C, D, AD, BC, BD, CD, A2, B2, C2, and D2 are statistically significant components of the model. The model can be improved by removing insignificant components from the model and leaving only the significant ones. AB, BD, and D2 components were removed from the model (Equation (3)).

Y = −200.082 + 5.98 A + 9.58 D + 4.96 C + 2.21 B − 0.051 AC − 0.066 AD + 0.051 BC + 0.038 CD − 0.37 A^2^ − 0.12 B^2^ − 0.35 C^2^ (3)

**Figure 2:**
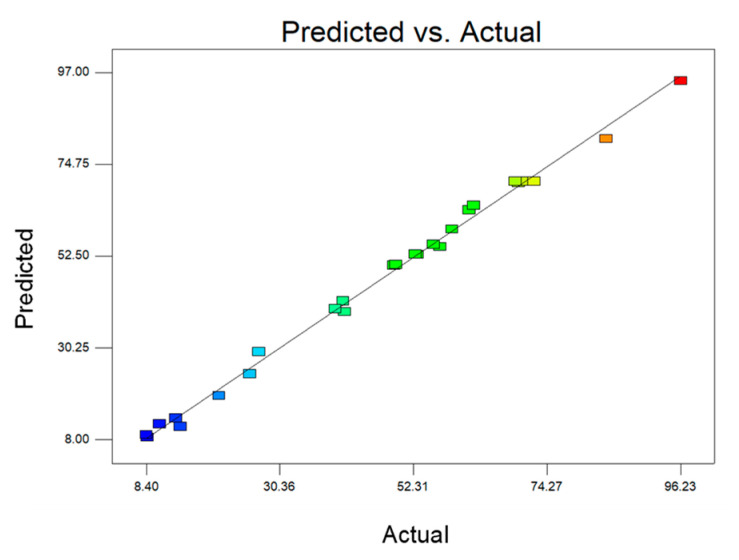
Correlation of actual and predicted values for transesterification yield.

The low value of the coefficient of variation (C.V. = 4.42%) indicates that the results of the fitted model are reliable. The quality of the model fit was assessed by the coefficient of determination (R^2^), which was calculated to be 0.9961. This means that 99.61% of the experimental data confirm the agreement with the data predicted by the model. The value of the adjusted coefficient of determination (R^2^adj) was 0.9930 and the value of the predicted coefficient of determination (R^2^_pred_) was 0.9844, indicating that the actual results agree with the predicted results (Figure 2).

This means that the model is accurate and reliable for predicting and analysing the degree of transesterification of biodiesel. The value of the adjusted coefficient of determination (R^2^_Adj._ = 0.9930) is also very high, which confirms the significance of the model. A high value of predicted R^2^ (0.9844) indicates that the fitted model is reasonably accurate. The corresponding precision value is 61.803 for the model. This value represents the signal-to-noise ratio. A value greater than 4 is desirable. For this model, the corresponding precision value is more than 15 times the desired value. In this study, a ratio greater than 4 for adequate precision observed in all models validates that the model has an adequate signal, which indicates that the model can be used to navigate the design space. This was further supported by Figure 2, which shows that all the experimental values were scattered around the predicted values. As shown in Figure 2, the predicted values of transesterification yield obtained from the model and the actual experimental data were in good agreement.

**Figure 3:**
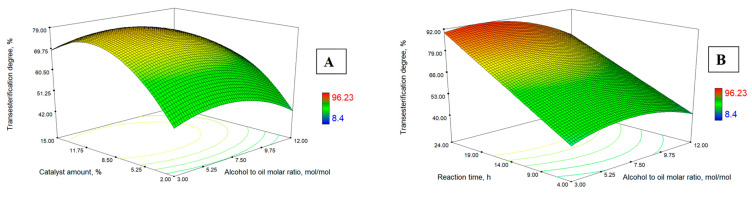

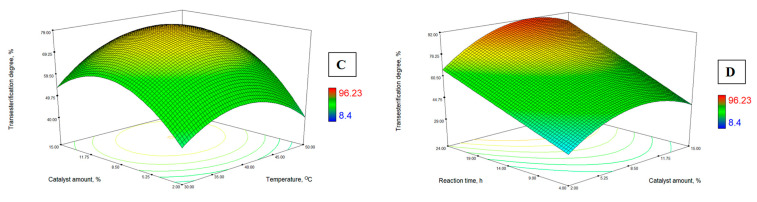
Response surface contour plot for the interaction between: (**A**)—the molar ratio of alcohol to oil and catalyst amount, (**B**)—the molar ratio of alcohol to oil and reaction time, (**C**)—the temperature and catalyst amoun, (**D**)—the catalyst amount and reaction time.

Based on the data of the initial analysis, three-dimensional (3-D) contour plots were compiled to assess the best conditions for the transesterification degree, as shown in Figure 3A–D. Each figure examines the patterns of process response (transesterification degree), determining the relationships between the response and independent variables (amount of enzyme, the molar ratio of alcohol to oil, temperature, and duration). The values of the dependent variables are selected in such a way as to obtain the maximum response value, and independent variables are fixed (on the X and Y axes). Figure 3 shows the effect of the transesterification degree on: Figure 3A—the molar ratio of alcohol to oil and the amount of the biocatalyst; Figure 3B—the molar ratio of alcohol to oil and the duration of the reaction; Figure 3C—temperature and the amount of the biocatalyst; Figure 3D—the amount of biocatalyst and the duration of the reaction.

From the data presented, it can be seen that the content of the biocatalyst had a positive effect on transesterification degree (Figure 3A). With an increase in the amount of biocatalyst to 11%, the transesterification degree increased. With a further increase in the amount of biocatalyst, it was noted that the transesterification degree decreases. This could be explained by the fact that the higher amount of immobilised enzyme increased the viscosity of the reaction medium it and therefore created conditions for the less effective transfer of the substrate to the active sites of enzyme particles [33]. The catalytic performance of lipase is affected by the effective interfacial area [34]. In the case when accession of substrates to the active sites of the excess enzymes, the interfacial area was reduced and transesterification degree was decreased, despite the fact that more immobilized enzyme was added. The results given in Figure 3 show that, when assessing the transesterification degree depending on the reaction time, temperature, and molar ratio, the maximum yield is always obtained at a concentration of about 11% of the lipase from the oil content. This is unlike Marín-Suárez et al., who studied the use of fish oil transesterification with ethanol using three commercial immobilized enzymes: Lipozyme^®^ RM IM, Lipozyme^®^ TL IM, and Novozym^®^ 435 [13]. They received a maximum biodiesel yield of around 75% at a Lipozyme^®^ RM IM content of 50% [13]. Ramakrishnan et al. studied the transesterification of salmon oil with methanol and obtained the highest biodiesel yield using 15% Novozym^®^ 435 (immobilized lipase from Candida antartica) [18].

Figure 3B shows the effect of the molar ratio of alcohol to oil and the duration of the reaction on the transesterification degree. The molar ratio of oil to alcohol is essential for obtaining high amount of alkyl esters [35]. As scientists observed, the amount of alcohol used for the conversion to biodiesel should be slightly higher than the stoichiometric content, equal to the number of fatty acids in the oil, to compensate for thermodynamic or kinetic restrictions, i.e., a higher alcohol content is used to push the balance to the right in order to obtain a higher yield of alkyl esters. From the data presented, it can be seen that the molar ratio of isoamyl alcohol to salmon oil has a significant influence on the transesterification degree (*p* < 0.0001 (Table 4)). With an increase in the molar ratio of alcohol to oil from 3:1 to 6:1, the transesterification degree increases. This ratio also depends on the type of raw material used [18,35,36]. However, with an increase in the molar ratio higher than 6:1, the transesterification degree slightly decreased. This could be explained by the fact that an excess of isoamyl alcohol can inhibit the activity of the enzyme [37].

The reaction temperature affects the activity and stability of enzymes, as well as influences the transesterification reaction rate. In addition, any increase in the solubility of the substrate depends on temperature, which can lead to an increase in the interaction of the enzyme and the substrate [37]. Foreign scientists have found that the optimum temperature for the synthesis of biodiesel using various lipases is from 30 to 55 °C [18,21,38]. At this temperature, lipases have an activity of more than 90% [30]. The results of our tests at a temperature of 30–50 °C confirm this observation. In the Figure 3C, presented data show that, by increasing the temperature from 30 °C to 45 °C, the transesterification degree increases. A higher temperature has a negative effect, since the efficiency of transesterification is reduced. This result can be explained by the low resistance of the biocatalyst to high temperatures. Using the enzyme preparation Lipozyme^®^ RM IM, other scientists have noticed that the most effective temperatures for this enzyme are 40–45 °C [37,39]. The research results showed that high temperatures lead to the denaturation of lipase.

A longer duration of the reaction increased the transesterification degree of salmon oil with isoamyl alcohol (3-d). In this study, the duration of the process ranged from 4 h to 24 h. The observed trend indicates that a high transesterification degree is obtained at the maximum duration of the process. The results obtained by other scientists for the influence of duration on the ester yield are contradictory. Some claim that the process rate is relatively high. Moreira et al. [30] studied the transesterification of babassu oil (residual babassu oil) (*Orbignya* sp.) with ethanol, using the enzyme preparation Novozym^®^ 435, and found that the maximum yield of biodiesel is obtained in 2 h. Meanwhile, researchers Park et al. [32] in the salmon oil transesterification with methanol reaction using lipase Lipozyme^®^ RM IM reached their peak biodiesel yield in 6 h, while scientists Ramakrishnan et al. [18], in the salmon oil methanolysis reaction using Novozym^®^ 435 as a catalyst, reached their highest biodiesel yield in 16 h. Deng et al. [40], for sunflower oil transesterification with ethanol, propanol, and butanol, used the same catalyst Lipozyme^®^ RM IM and reached the maximum biodiesel yield in 24 h, while researchers Amin et al. [41], in the methanolysis reaction of sweet basil seed oil using the biocatalyst Novozym^®^ 435, reached the highest biodiesel yield in 72 h.

### 3.4. Determination of Optimal Conditions and Their Validation

According to the prediction of the regression equation, it was determined that the maximum transesterification degree of 88.45% is obtained at the molar ratio of alcohol to oil 6:1, 11% of biocatalyst (based on the mass of oil), temperature 45 °C, and 4 h. for reaction time. The purpose of this experiment was not to predict the maximum transesterification degree, but to find the optimal reaction condition under which the maximum transesterification degree can be obtained in the shortest reaction time, using the least amount of alcohol and biocatalyst. The results of the statistical analysis were verified experimentally to validate the analysis. A regression equation was used to predict the transesterification degree using one selected set of parameters. The values of predicted and actual transesterification degree are shown in Table 5. The actual data presented are the average of three replicate experiments. It is clear that the obtained experimental results agree with the predicted results with the smallest error, which confirms the prediction of the developed regression equation.

### 3.5. Gradual Addition of Isoamyl Alcohol

Although an excess of alcohol facilitates the mixing of the mixture system, a high alcohol-to-oil molar ratio increases the polarity of the reaction medium, which is often associated with biocatalyst inactivation. Therefore, in order to avoid the negative effect of high concentration of alcohol on lipase, due to which lipase is inactivated, the experiments of gradual addition of isoamyl alcohol to the reaction mixture were performed. Based on optimized biodiesel synthesis conditions, the efficiency of the stepwise addition of isoamyl alcohol to further increase the transesterification degree was evaluated. Instead of using the optimal determined amount of isoamyl alcohol at 6:1 molar ratio of alcohol to oil, the experiment was started at 2:1 molar ratio of isoamyl alcohol to oil for the first hour and then at 2:1 molar ratio of isoamyl alcohol to oil for the next 2 h. Other portions of isoamyl alcohol were added every 1 h during the reaction. A transesterification degree of 96.5% was obtained in this experiment, i.e., about 10% higher than the results obtained with a single addition of full amount of isoamyl alcohol. The higher transesterification degree could be explained by higher activity of the enzyme preparation, probably because the arrangement of the enzyme molecules on the carrier facilitated the access of the substrate to the active centres of the lipase. In this case, the alcohol had a positive effect on the reaction kinetics, facilitating the formation of a homogeneous suspension of reactants and biocatalysts [42]. Similar results were obtained by other researchers [18,36,38] when, during two or three stepwise additions of alcohol to the transesterification medium, the yield of biodiesel was increased up to 10% when methanol and ethanol were used as acyl acceptors. When using higher alcohols (propanol, isobutanol, 1-butanol), Deng et al. [40] increased the yield of biodiesel over four times when alcohol was gradually introduced into the reaction media.

### 3.6. Determination of Optimal Conditions and Their Validation

The properties of the produced biodiesel are presented in Table 6. The physicochemical properties of biodiesel were determined according to the EN 14214 standard, and the determined properties were compared with the requirements of biodiesel and mineral diesel standards (ASTM D6751, 14214, and EN 590).

As can be seen from Table 6, the obtained values correspond to biodiesel standards EN 14214 and ASTM 6751. Compared to the requirements for mineral diesel, the produced biodiesel had a higher viscosity and density. Kinematic viscosity and density are the most important properties of fuel that affect diesel engine performance and emissions. Due to the higher viscosity and density, the fuel becomes larger droplets, is poorly sprayed, does not burn completely, and increases NOx emissions [43]. The viscosity (4.93 ± 0.014 mm^2^/s) and density (880.67 ± 0.23 kg/m^3^) of produced biodiesel met the requirements of ASTM 6751 and EN 14214 specifications and can be used in a diesel engine as an alternative fuel.

Calorific value is an important parameter for fuel selection. Biodiesel standards ASTM D6751 and EN 14214 do not specify the calorific value, but EN 14213 (biodiesel for heating) specifies the minimum value of 35 MJ/kg [44]. In our tests, the calorific value of produced biodiesel was about 40.03 ± 0.15 MJ/kg. However, the calorific value of standard mineral diesel is 42.54 MJ/kg, i.e., about 6% higher than biodiesel. The reason for the lower calorific value of biodiesel is the presence of chemically bound oxygen in biodiesel, which reduces the calorific value of fatty acid esters by about by 6% compared with mineral diesel.

## 4. Conclusions

Waste salmon oil has great potential as a sustainable source for biodiesel production. The immobilized enzyme Lipozyme^®^ RM IM is an effective biocatalyst for the synthesis of isoamyl alcohol esters of salmon oil. After studying the effectiveness of three immobilized industrial lipases (Lipozyme^®^ RM IM, Lipozyme^®^ TL IM, Lipozyme^®^ 435) in the process of transesterification of salmon oil with isoamyl alcohol, it was found that Lipozyme^®^ RM IM has the highest efficiency and can be used as enzymatic catalyst for biodiesel synthesis. The transesterification process was influenced by four independent variables: molar ratio of isoamyl alcohol to oil; temperature; reaction time; and amount of biocatalyst. Using response surface analysis, the interaction of independent variables was investigated, and the following optimal parameters for transesterification of salmon oil with isoamyl alcohol were determined: 11% enzyme concentration; 45 °C; 4 h; and 6:1 alcohol-to-oil molar ratio. The transesterification degree at these conditions was 87.23%. The results of the statistical analysis were verified experimentally to validate the analysis results. The obtained experimental results agreed with the predicted results.

In order to increase the transesterification degree and avoid poisoning of the enzyme preparation with excess of alcohol, the possibilities of gradual addition of isoamyl alcohol to the reaction medium were studied. It was found that the degree of transesterification can be increased to 96.5% by gradually adding amount of isoamyl alcohol, equal to the 2:1 molar ratio of alcohol and oil, to the reaction medium every hour.

The physical and chemical properties of the product obtained during the transesterification process of salmon oil with isoamyl alcohol were compared with the main requirements of the standards defining the physical and chemical parameters of biodiesel and mineral diesel: EN 14214, ASTM 6751, and EN 590. Esters of salmon oil and isoamyl alcohol have been found to meet the requirements for conventional biodiesel used in the transport sector. Compared to the requirements for mineral diesel, the salmon oil transesterification product had a higher viscosity and density. In addition, the studied biodiesel had a lower calorific value than mineral diesel.

## Figures and Tables

**Table 1:** Independent variables and levels used for CCD in transesterification of salmon oil with isoamyl alcohol.

Factors	Name	Units	Low Actual	High Actual
A:	Alcohol-to-oil molar ratio	mol/mol	3	10
B:	Reaction temperature	°C	30	50
C:	Amount of catalyst (from oil mass)	%	2	15
D:	Process duration	h	4	24

**Table 2:** Central composite design matrix and observed and modelled results.

No	Alcohol-to-Oil Molar Ratio, mol/mol	Reaction Temperature, °C	Amount of Catalyst, % (from Oil Mass)	Process Duration, h	Transesterification Degree, %	
					Experimental	Predicted
1	3.00	30.00	2.00	4.00	14.01	11.23
2	12.00	30.00	2.00	4.00	10.54	11.83
3	3.00	50.00	2.00	4.00	8.57	8.62
4	12.00	50.00	2.00	4.00	8.40	9.22
5	3.00	30.00	15.00	4.00	20.36	18.68
6	12.00	30.00	15.00	4.00	13.28	13.29
7	3.00	50.00	15.00	4.00	26.93	29.37
8	12.00	50.00	15.00	4.00	25.40	23.98
9	3.00	30.00	2.00	24.00	52.99	52.91
10	12.00	30.00	2.00	24.00	40.72	41.64
11	3.00	50.00	2.00	24.00	49.08	50.30
12	12.00	50.00	2.00	24.00	40.97	39.02
13	3.00	30.00	15.00	24.00	69.57	70.25
14	12.00	30.00	15.00	24.00	52.66	52.98
15	3.00	50.00	15.00	24.00	84.00	80.94
16	12.00	50.00	15.00	24.00	61.52	63.67
17	2.10	40.00	8.50	14.00	62.21	64.80
18	12.90	40.00	8.50	14.00	56.66	54.80
19	7.50	28.00	8.50	14.00	49.47	50.50
20	7.50	52.00	8.50	14.00	55.64	55.34
21	7.50	40.00	0.70	14.00	39.44	39.79
22	7.50	40.00	16.30	14.00	58.66	59.04
23	7.50	40.00	8.50	2.00	32.52	*
24	7.50	40.00	8.50	26.00	96.23	95.00
25	7.50	40.00	8.50	14.00	70.48	70.59
26	7.50	40.00	8.50	14.00	72.12	70.59
27	7.50	40.00	8.50	14.00	69.08	70.59

*A diagnostic test for externally studentized errors helped detect data that were poorly described by the model. These measurements were excluded from the analysis.

**Table 3:** Properties of salmon oil.

Parameter	Value
Acid value, mg KOH/g	14.56 ± 0.23
Acidity, %	7.64 ± 0.42
Moisture content, %	3.1 ± 0.05
Iodine value, g L_2_/100 g	122.92 ± 0.95
Density at 15 °C, kg/m^3^	930 ± 2.00
Viscosity at 40 °C, mm^2^/s	37.42 ± 0.21
a* colour value	−1.74 ± 0.03
b* colour value	3.88 ± 0.02
L* colour value	3.79 ± 0.05
Saturated fatty acids, %:	20.26
Butyric acid (C4:0)	0.02 ± 0.0009
Caproic acid (C6:0)	0.06 ± 0.01
Caprylic acid (C8:0)	0.23 ± 0.05
Capric acid (C10:0)	0.29 ± 0.07
Undecylic acid (C11:0)	0.04 ± 0.0002
Lauric acid (C12:0)	0.04 ± 0.0003
Tridecanoic acid (C13:0)	1.46 ± 0.15
Myristic acid (C14:0)	2.63 ± 0.13
Pentadecanoic acid (C15:0)	0.2 ± 0.015
Palmitic acid (C16:0)	9.7 ± 1.78
Margaric acid (C17:0)	0.13 ± 0.045
Stearic acid (C18:0)	2.66 ± 0.26
Arachidic acid (C20:0)	1.07 ± 0.026
Heneicosylic acid (C21:0)	0.89 ± 0.033
Behenic acid (C22:0)	0.76 ± 0.05
Tricosylic acid (C23:0)	0.03 ± 0.0013
Lignoceric acid (C24:0)	0.05 ± 0.001
Unsaturated fatty acids, %:	79.76
Monounsaturated fatty acids, %:	52.92
Myristoleic acid (C14:1 cis-9)	0.41 ± 0.05
Palmitoleic acid (C16:1 cis-9)	2.86 ± 0.23
Heptadecenoic acid (C17:1 cis-10)	0.23 ± 0.044
Oleic acid (C18:1)	41.05 ± 0.58
Paullinic acid (C20:1)	3.85 ± 0.046
Erucic acid (C22:1)	2.92 ± 0.65
Nervonic acid (C24:1)	1.61 ± 0.25
Poliunsaturated fatty acids, %:	26.83
Linoleic acid (C18:2)	14.21 ± 0.95
Linolenic acid (C18:3)	5.28 ± 0.45
Eicosadienoic acid (C20:2)	0.04 ± 0.0001
Dihomo-gamma-linolenic acid (C20:3 8,11,14)	0.04 ± 0.0002
Eicosatrienoic acid (C20:3 11,14,17)	0.41 ± 0.11
Arachidonic acid (C20:4)	0.2 ± 0.05
Eicosapentaenoic acid (C20:5)	3.18 ± 0.47
Brassic acid (C22:2)	0.02 ± 0.001
Docosahexaenoic acid (C22:6)	3.45 ± 0.33
Oil molecular mass, g/mol	878.45 ± 2.05

**Table 4:** Analysis of variance of quadratic model (ANOVA).

Source of Variation	Sum of Squares	Degrees of Freedom (df)	Mean Squares	F Value	*p*-Value Prob > F	
Model	14,964.06	11	1360.37	321.38	<0.0001	significant
A-molar ratio	327.89	1	327.89	77.46	<0.0001	
B-temperature	77.06	1	77.06	18.21	0.0008	
C-catalyst	1215.76	1	1215.76	287.21	<0.0001	
D-duration	7109.11	1	7109.11	1679.47	<0.0001	
AC	35.94	1	35.94	8.49	0.0113	
AD	141.13	1	141.13	33.34	<0.0001	
BC	177.02	1	177.02	41.82	<0.0001	
CD	97.71	1	97.71	23.08	0.0003	
A^2^	293.37	1	293.37	69.31	<0.0001	
B^2^	786.79	1	786.79	185.87	<0.0001	
C^2^	1129.88	1	1129.88	266.93	<0.0001	
Residual	59.26	14	4.23			
Lack of Fit	54.63	12	4.55	1.97	0.3862	not significant
Pure Error	4.63	2	2.32			
Cor Total	15,023.32	25				
C.V. % = 4.42		R^2^ = 0.9961		Adeq Precision = 61.803		
R^2^_Adj_ = 0.9930		R^2^_Pred_ = 0.9844				

**Table 5:** Predicted and experimental degree of transesterification.

Alcohol-to-Oil Molar Ratio, mol/mol	Temperature, °C	Amount of Catalyst, % (from Oil Mass)	Duration, h	Transesterification Degree, %	
				Predicted	Actual
6.00	45.00	11.00	4.00	88.45	87.23 ± 0.03

**Table 6:** Properties of biodiesel and requirements of standards.

Properties	Unit	EN 14214	ASTM 6751	EN 590	Produced Biodiesel
Ester content	% (m/m)	Min 96.5			96.5 ± 2.05
Density at 15 °C	kg/m^3^	860–900	860–900	820–845	880.67 ± 0.23
Viscosity at 40 °C	mm^2^/s	3.5–5	1.9–6	2.0–4.5	4.93 ± 0.014
Acid value	mg KOH/g	0.5 max	0.5 max		0.41 ± 0.12
Iodine value	g L_2_/100 g	120 max			98.04 ± 1.02
Free glycerol	% (m/m)	0.02 max	0.02 max		0.004 ± 0.001
Monoglyceride content	% (m/m)	0.8 max			0.36 ± 0.02
Diglyceride content	% (m/m)	0.2 max			0.02 ± 0.001
Triglyceride content	% (m/m)	0.2 max			0.02 ± 0.001
Total glycerol	% (m/m)	0.25 max	0.24 max		0.011 ± 0.001

## Data Availability

Not applicable.
